# Maternal sciatic nerve administered bupivacaine induces hippocampal cell apoptosis in offspring

**DOI:** 10.1186/s12871-020-01143-2

**Published:** 2020-09-07

**Authors:** Alireza Mirkheshti, Alireza Shakeri, Elham Memary, Mansoureh Baniasadi, Jalal Zaringhalam, Ardeshir Tajbakhsh, Marzieh Mirzaei, Elena Lak

**Affiliations:** 1 Department of Anesthesiology, Shahid Beheshti University of Medical Sciences, Tehran, 1985717443 Iran; 2 Department of Physiology, School of Medicine, Shahid Beheshti University of Medical Sciences, Tehran, Iran; 3 Department of Gynecology, Tehran Azad University, Tehran, Iran; 4 Department of Gastroenterology, Shahid Beheshti University of Medical Sciences, Tehran, Iran

**Keywords:** Bupivacaine, Apoptosis, Akt, Pregnancy

## Abstract

**Background:**

Bupivacaine, an amid-type local anesthetic, is widely used for clinical patients especially in pregnant women. In addition to neurotoxicity effect of bupivacaine, it can cross the placenta, accumulates in this tissue and retained in fetal tissues. Nevertheless, whether bupivacaine can cause neurotoxicity in fetus remains unclear. Hence, this study was design to investigate the effects of maternal bupivacaine use on fetus hippocampal cell apoptosis and the possible related mechanism.

**Methods:**

On day 15 of pregnancy, sciatic nerve of pregnant wistar rat (180–200 g) were exposed by lateral incision of the right thigh and 0.2 ml of bupivacaine was injected. After their delivery, we randomly selected one male offspring of every mother. On day 30 after of their birth, the rat’s hippocampi were isolated for molecular studies. Western blotting was used to examine the expression of cleaved caspase-3, caspase-8 and p-Akt in fetal hippocampus.

**Results:**

Our results showed that maternal bupivacaine use caused a significant increment of cleaved caspase-3 and caspase-8 expression in fetal hippocampus compared with the sham group. In addition, maternally administered bupivacaine could significantly decrease hippocampal P.Akt/T.Akt ratio which was concurrent with an increment of cleaved caspase-3 and caspase-8 expression.

**Conclusion:**

Our data suggest that maternal bupivacaine use increases fetal hippocampal cell apoptosis markers such as caspase 8 and cleaved caspase 3, at least in part, via inhibiting the Akt activation.

## Background

Bupivacaine, an amid-type local anesthetic, is widely used for spinal and epidural anesthesia, peripheral nerve blockade, sympathetic nerve block and postoperative analgesia in clinical patients, especially in pregnant patients by providing excellent sensory anesthesia [1, 2]. However, local anesthetics may have potential neurotoxicity and induce nonreversible neurological complications [1, 3–5]. In this regard, a growing body of data indicates that bupivacaine triggers a complex cascade response leading to neuronal apoptosis [6–9]. It has been reported that disruption of calcium homeostasis, reduced mitochondrial membrane potential, ROS generation and DNA damage in the neuronal population are implicated in the pathogenesis of bupivacaine-induced neurotoxicity [3, 10, 11]. The exact mechanisms by which bupivacaine induces apoptosis have not been elucidated entirely. However, different studies have reported that several signaling pathways such as the PERK [12, 13], IRE1 [3], glycogen synthase kinase-3 (GSK3) [14], MAPK [15, 16] and Akt [4, 12] might be responsible for bupivacaine- induced apoptosis.

Although the placenta is proposed as a protective barrier, there are mechanisms of drug transport across this tissue that expose the fetus to drugs taken by pregnant women [17–19]. It has been a general finding that the placenta does not limit the fetal transfer of local anesthetics [20–22]. By using a human placental model in rats, it is revealed that bupivacaine accumulates in the placenta and is retained in fetal tissues [22, 23]. Some animal studies have shown that bupivacaine has an adverse effect on the fetus so, according to the US FDA, bupivacaine is a pregnancy category C drug. While, based on the Australian categorization system for prescribing medicines in pregnancy, bupivacaine is a category A drug that is widely used in pregnant women and has no reported direct or indirect harmful effects on the fetus [2, 22]. Because of controversial reports, further research is required to determine the possible neurotoxic potency of maternal bupivacaine use on the fetus and newborn. Therefore, our main purpose in the present study was to investigate the effects of maternal bupivacaine use on fetus hippocampal apoptosis and the possible related mechanism.

## Methods

### Laboratory animals and experimental procedures

All procedures were approved by the ethics committee of Shahid Beheshti University of Medical Sciences (IR.SBMU.RETECH.REC.1398.031) which followed Guidelines of ethical standards for the care and use of laboratory animals for animal research [24]. The animals were obtained from Laboratory Animal Center, Shahid Beheshti University of Medical Sciences. They were housed in polypropylene cages under standard environmental conditions (22 ± 2  °C, humidity 60–70%, and 12 h light/dark cycle) and allowed standard water and food intake. Adult female Wistar rat weighting 180–200 g was used for mating. Pregnant rats on day 15 of pregnancy randomly divided into three groups as follows (a) Control group (sciatic nerve was exposed but no drug was administered), (b) Sham+ vehicle group (sciatic nerve was exposed and saline was injected) and (c) Bupivacaine group (sciatic nerve was exposed and blocked with bupivacaine administration) (*n* = 6 / each group).

### Surgery and drug administration

On day 15 of pregnancy, the pregnant rats became anesthetized with intraperitoneal injection of Ketamine 100 mg/kg and Xylazine 10 mg/kg mixture. Then, the sciatic nerve of the rat’s right thigh was isolated and based on previous studies [25, 26], 0.2 ml of Bupivacaine 0.5% (Astra Zeneca, Austria), in Bupivacaine group, and 0.2 ml of Normal Saline 0.9%, in the Sham+vehicle group, was injected beneath the clear fascia surrounding the sciatic nerve but outside the perineurium. After performing nerve block, we sutured the muscles and skin by Vicryl 6–0 and Nylon 4–0 sutures. After recovery, all rats were returned to their places and were cared at the time of their delivery. Then, we randomly selected one male offspring of every mother and kept under standard condition until the 30th day after of their birth. All offspring in control, sham and bupivacaine group have no significant difference in birth weight and weight gain during the study. In day 30, the rats (*n* = 6 per each group) were anaesthetized by CO_2_ inhalation then decapitated. The hippocampi were immediately isolated on the ice and kept in liquid nitrogen for 24 h and then stored in − 80 °C until molecular analysis. All animals used in the present study, finally were anaesthetized by CO2 inhalation then scarified by decapitation.

### Western blotting

The hippocampi were homogenized in the cold RIPA lysis buffer (50 mM Tris-HCl, pH 8.0; 150 mM NaCl; 1% Triton X-100; 0.5% Na-Deoxycholate; 0.1% SDS (sodium dodecyl sulfate)) supplemented with protease and phosphatase inhibitors cocktail (was purchased from Pierce). Bradford method was used to quantify the protein content of each sample. Then, the equal amounts of proteins (40 μg) were separated using 12% polyacrylamide gel electrophoresis, transferred to a activated PVDF membrane (Roche Diagnostics, Indianapolis, IN, USA), blocked with blocking buffer (5% BSA) 1 h in room temperature and incubated at 4 °C overnight with primary anti-rabbit antibodies against phospho-Akt (Ser473) (1:3000, cell signaling #4060), Akt (1:3000, cell signaling #4685), caspase-3 (1;3000, ab184787), caspase-8 (1;3000, cell signaling #4790) and β-actin (1:15000, cell signaling #4970). After washing with TBST, the membranes incubated for 1:30 h with secondary HRP-conjugated anti-rabbit antibody (1:20000, cell signaling #7074) at room temperature, visualized by Amersham ECL select Western Blotting Detection Kit (RPN2235), and exposed to radiography films (Kodak). All membranes were stripped and incubated with primary antibody against β-actin. Finally, the radiographic films were scanned and blot quantification of protein bands density was calculated by Image-J software.

### Statistical analysis

Statistical analyses of data and drawing charts were performed using the GraphPad Prism 7.01. For comparison of variables between the groups, one-way analysis of variance (ANOVA) followed by Post hoc Tukey’s test was used. All data have been shown as means ± S.E.M. In all statistical comparisons, *P* < 0.05 is considered as a significant difference.

## Results

### Maternal bupivacaine administration increases the level of fetal hippocampal cleaved caspase-3

**Fig. 1:**
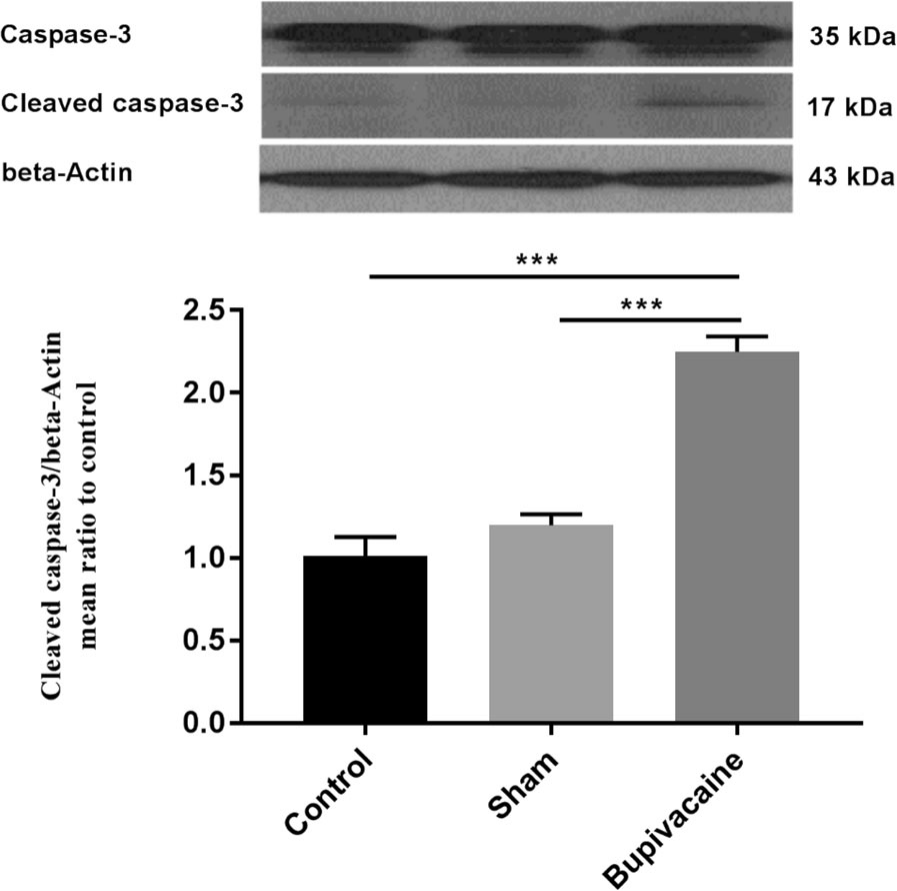
Maternally administered bupivacaine, significantly increase cleaved caspase-3 expression in the fetal hippocampus. Representative cropped western blot of cleaved caspase-3 (19 kDa) which is normalized to beta-Actin. Data are represented as mean ± SEM (*n* = 6 rats/group). ***P < 0.001: comparison of cleaved caspase-3 protein band intensity between different groups. Full-length blots are presented in Supplementary Fig. 1

Activation of caspase-dependent apoptosis was assessed through western blot analysis of cleaved caspase-3. Caspase-3 play a main role in apoptosis and its cleavage represents its activation [27]. There was no significant difference in caspase-3 activity between the control and sham group while, maternal bupivacaine use caused a significant increase in cleaved caspase-3 expression in the fetal hippocampus compared with the sham group (*P* < 0.001) (Fig. 1).

### Overexpression of fetal hippocampal caspase-8 after maternal bupivacaine use

**Fig. 2:**
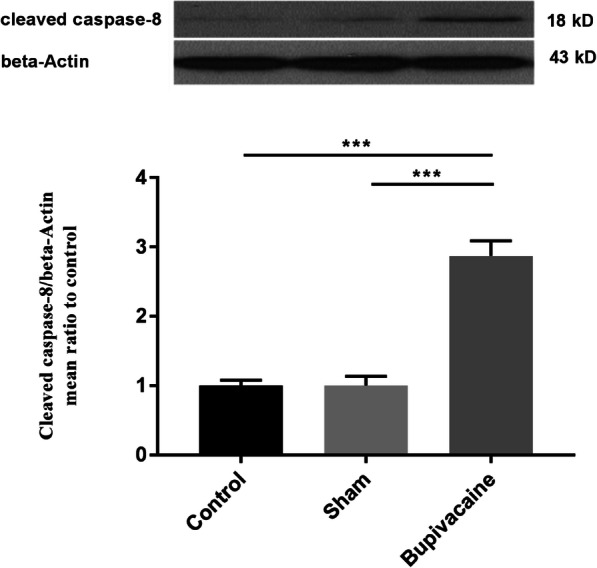
Maternal bupivacaine usage, significantly increased cleaved caspase-8 expression in the fetal hippocampus. Representative cropped western blot of cleaved caspase-8 (18 kDa) which is normalized to beta-Actin. Data are represented as mean ± SEM (*n* = 6 rats/group). ***P < 0.001: comparison of cleaved caspase-8 protein band intensity between different groups. Full-length blots are presented in Supplementary Fig. 2

To test the role of maternal bupivacaine use on fetal hippocampal cell apoptosis, caspase-8 expression was also assessed by western blotting. The data showed that there was no significant difference in caspase-8 expression between the control and sham groups. One- way ANOVA followed by Tukey’s test showed that maternal bupivacaine use could increase hippocampal caspase-8 expression in the bupivacaine group compared with the sham group (*P* < 0.001) (Fig. 2).

### Phosphorylation of Akt decreases after maternal bupivacaine use

**Fig. 3:**
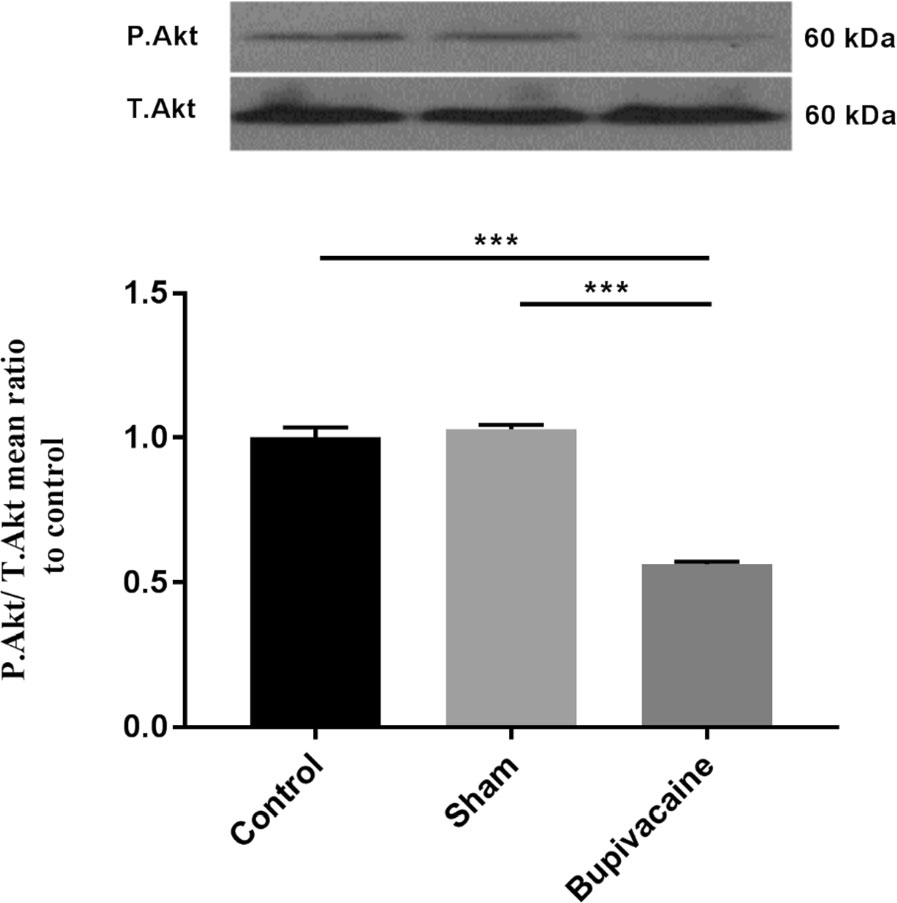
Maternal administration of bupivacaine significantly decreased Akt activity in fetal hippocampus. Representative cropped western blot of P. Akt (60 kDa) which is normalized to T.Akt. Data are represented as mean ± SEM (*n* = 6 rats/group). ***P < 0.001: comparison of P. Akt protein band intensity between different groups. Full-length blots are presented in Supplementary Fig. 3

PI3K/Akt pathway plays a regulatory role in different biological processes such as proliferation and cell survival [28–30]. Western blot was done to investigate the effect of maternal bupivacaine use on fetal hippocampal P.Akt/T.Akt ratios. Our results revealed that maternal bupivacaine use significantly decreased hippocampal P.Akt/T.Akt ration in the bupivacaine group compared with sham group (*P* < 0.001) (Fig. 3).

## Discussion

Pregnancy is a unique experience in the lifetime of women. The mother’s health is directly connected to the fetus’s health. Therefore, it is essential to maintain the health of pregnant women. Since, local anesthetics are commonly used for surgical procedures in the pregnant women [31–33], it is necessary to understand the effects of maternally administered local anesthetics on the fetus. For this purpose, this study aimed to elucidate the adverse effects of maternal bupivacaine use on fetus hippocampal cell apoptosis and the possible related mechanism. To assess the activation of caspase-dependent apoptosis pathways in fetal hippocampal following maternally administered bupivacaine, we measured activation of caspase-3 and caspase-8. It is revealed that caspase-3 play a key role in apoptosis and its cleavage represents its activation [27, 34]. Caspase-3 is activated during both extrinsic and intrinsic apoptosis pathways [27, 35]. Also, caspase-8 is the initiator caspase that play a critical role in the extrinsic apoptotic signaling pathway. Our result revealed that maternal bupivacaine use could increase apoptosis-related proteins, cleaved caspase-3 and caspase- 8 expressions in the hippocampal of the fetus compared with the sham group.

A growing body of researches in the last years, both laboratory and clinical settings, reported that, although happened in rare-event situations, local anesthetic reagents, like bupivacaine, ropivacaine, lidocaine and mepivacaine, might induce severe neurological injury in both animal and humans [14, 36–38]. In this regard, Yu et al. (2017) shown that in neuronal population, bupivacaine could significantly increase apoptosis and induce much severe neurotoxicity than other local anesthetics, such as mepivacaine or procaine [10]. In addition, another study showed that bupivacaine could induce neural apoptosis and neurite degeneration in DRG neurons [11]. Available evidences shows that local anesthetics have systemic absorption, and placenta does not limit the fetal transfer of maternally administered amide-linked local anesthetics, such as bupivacaine [19, 20, 39]. It is revealed that bupivacaine crosses the placenta, accumulates in this tissue and is retained in fetal tissues [18, 21, 22]. Bupivacaine enters in the fetal liver through umbilical venous blood perfused in this organ. The 3-hydroxybupivacaine can remain detectable in the fetus liver up to 4 h. This suggesting that bupivacaine can metabolize in the fetal liver [22]. The fetus eliminates the bupivacaine by diffusing it into the maternal compartment through the placental membrane. Although the majority of the bupivacaine metabolites are more polar, and it is unlikely that the placental membrane crosses these metabolites back into the maternal compartment, possibly resulting in the accumulation of metabolites in various fetal tissues. The half of the fetal circulation directly reaches the heart and brain, thus it is possible that reduced fetal ability to remove drugs can cause prolonged adverse effects on these tissues [40, 41]. Although there are no adequate and well-controlled studies about the adverse effect of maternal bupivacaine on the fetus, our results for the first time suggest that, maternally administered bupivacaine, could have an adverse effect on fetal brain and induce hippocampal cell apoptosis.

The exact mechanism by which bupivacaine induces apoptosis have not been elucidated entirely. However different studies have reported that several signaling pathways, such as PERK [12, 13], IRE1 [3], GSK3 [14], MAPK [15, 16] and Akt [4, 12], might be responsible for bupivacaine- induced apoptosis. In exploring the signaling mechanism; we focused on Akt, which is a well-known anti-apoptosis molecule. Our results showed that maternal bupivacaine use markedly decreases the phosphorylation levels of Akt. A number of studies have demonstrated that Akt, a key kinase downstream of the PI3-kinase, plays a crucial role in cell survival and death pathway of neurons [12, 29, 42, 43]. Recent studies have confirmed that Akt-signaling pathway involves in the bupivacaine-induced apoptosis in adults [44, 45]. In this regard, Fan et al. (2016) reported that bupivacaine-induced neurotoxicity in SH-SY5Y cells is mediated thorough inactivating Akt signaling pathway [1]. In consistent with previous studies, we observed that bupivacaine decreased the phosphorylation levels of Akt in the fetal hippocampus, which was concurrent with an increment of apoptotic markers.

## Conclusions

Taken together, our data suggest that maternally administered bupivacaine increases fetal hippocampal cell apoptosis markers such as caspase 8 and cleaved caspase 3, at least in concurrent with inhibition of the Akt activation.

## Supplementary information

**Additional file 1.**

## Data Availability

The datasets used and/or analyzed during the current study are available from the corresponding author on reasonable request.

## Abbreviations

Akt: Protein kinase B
P.Akt: Phosphorylated Akt
PI3K: Phosphatidylinositol 3-kinases
T.Akt: Total Akt
