# Supplementary material for: Maternal sciatic nerve administered bupivacaine induces hippocampal cell apoptosis in offspring
Source: BMC Anesthesiol. 2020 Sep 7;20:228. doi: 10.1186/s12871-020-01143-2 (PMC7487602; doi:10.1186/s12871-020-01143-2)
Supplement: Supplementary file 1 — Additional file 1. [file 12871_2020_1143_MOESM1_ESM.docx]

Supplementary Figure 1. The identified wells represent Caspase-3, Cleaved Caspase-3 and B-actin and other wells are related to another studies. The blots are cropped from the marked location with the down arrow.


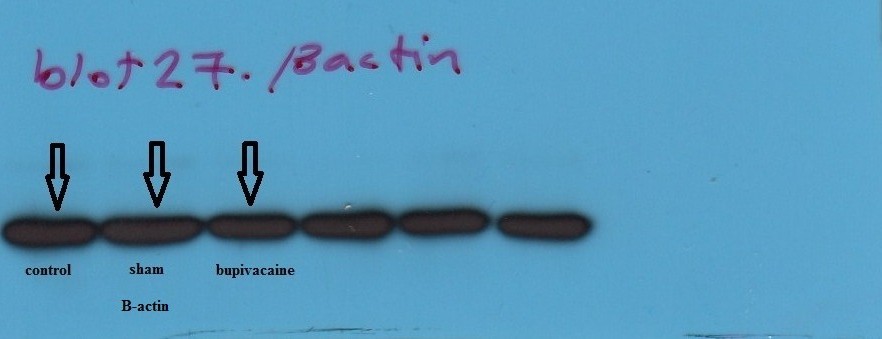

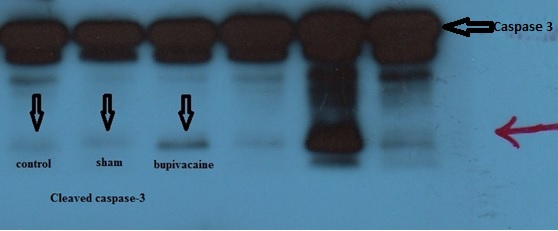

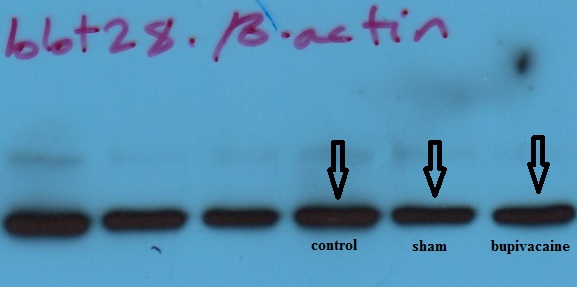

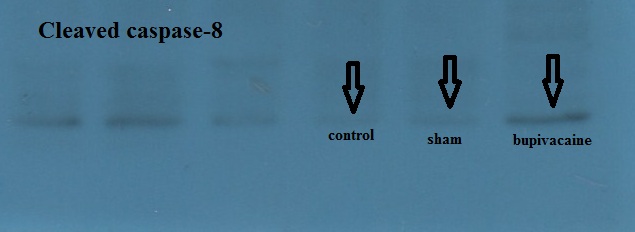


Supplementary Figure 2. The identified wells represent cleaved Caspase-8 and B-actin and other wells are related to another studies. The blots are cropped from the marked location with the down arrow.


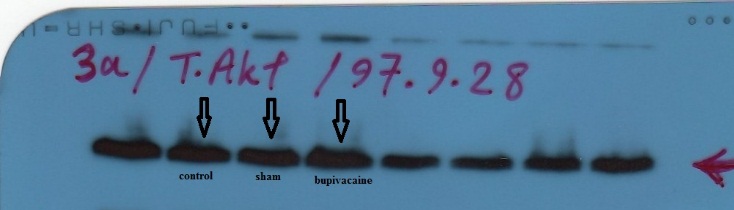

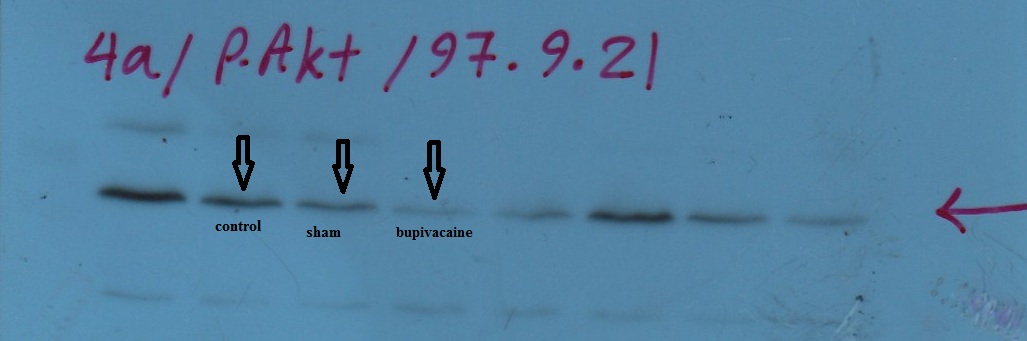


Supplementary Figure 3. The identified wells represent T.Akt and P.Akt and other wells are related to another studies. The blots are cropped from the marked location with the down arrow.
